# Bad apples or spoiled barrels? Multilevel modelling analysis of variation in high-risk prescribing in Scotland between general practitioners and between the practices they work in

**DOI:** 10.1136/bmjopen-2015-008270

**Published:** 2015-11-06

**Authors:** Bruce Guthrie, Peter T Donnan, Douglas J Murphy, Boikanyo Makubate, Tobias Dreischulte

**Affiliations:** 1 Quality, Safety and Informatics Research Group, Population Health Sciences Division, Medical Research Institute, University of Dundee, Mackenzie Building, Kirsty Semple Way, Dundee, UK; 2 Dundee Epidemiology and Biostatistics Unit, Population Health Sciences Division, Medical Research Institute, University of Dundee, Mackenzie Building, Kirsty Semple Way, Dundee, UK; 3 University of Dundee Medical School, Dundee, UK; 4 Faculty of Medicine, Department of Public Health, University of Botswana, Gaborone, Botswana; 5 NHS Tayside Medicines Governance Unit, Mackenzie Building, Kirsty Semple Way, Dundee, UK

**Keywords:** PRIMARY CARE, STATISTICS & RESEARCH METHODS, THERAPEUTICS

## Abstract

**Objectives:**

Primary care high-risk prescribing causes significant harm, but it is unclear if it is largely driven by individuals (a ‘bad apple’ problem) or by practices having higher or lower risk prescribing cultures (a ‘spoiled barrel’ problem). The study aimed to examine the extent of variation in high-risk prescribing between individual prescribers and between the practices they work in.

**Design, setting and participants:**

Multilevel logistic regression modelling of routine cross-sectional data from 38 Scottish general practices for 181 010 encounters between 398 general practitioners (GPs) and 26 539 patients particularly vulnerable to adverse drug events (ADEs) of non-steroidal anti-inflammatory drugs (NSAIDs) due to age, comorbidity or co-prescribing.

**Outcome measure:**

Initiation of a new NSAID prescription in an encounter between GPs and eligible patients.

**Results:**

A new high-risk NSAID was initiated in 1953 encounters (1.1% of encounters, 7.4% of patients). Older patients, those with more vulnerabilities to NSAID ADEs and those with polypharmacy were less likely to have a high-risk NSAID initiated, consistent with GPs generally recognising the risk of NSAIDs in eligible patients. Male GPs were more likely to initiate a high-risk NSAID than female GPs (OR 1.73, 95% CI 1.39 to 2.16). After accounting for patient characteristics, 4.2% (95% CI 2.1 to 8.3) of the variation in high-risk NSAID prescribing was attributable to variation between practices, and 14.2% (95% CI 11.4 to 17.3) to variation between GPs. Three practices had statistically higher than average high-risk prescribing, but only 15.7% of GPs with higher than average high-risk prescribing and 18.5% of patients receiving such a prescription were in these practices.

**Conclusions:**

There was much more variation in high-risk prescribing between GPs than between practices, and only targeting practices with higher than average rates will miss most high-risk NSAID prescribing. Primary care prescribing safety improvement should ideally target all practices, but encourage practices to consider and act on variation between prescribers in the practice.

**Strengths and limitations of this study**

- The study used data routinely recorded in general practitioner (GP) electronic medical records making it possible to examine actual practice on a large scale, and the data used came from practices participating in a national morbidity data set which had all received training and financial support to maintain particularly high quality data.
- We additionally examined data quality carefully, and restricted analysis to a topic (non-steroidal anti-inflammatory drugs (NSAID) prescribing) and type of prescription (initiation of NSAID prescribing) where we could demonstrate that data quality was high.
- All routine data studies share limitations, notably the type and quality of the data recorded, although our choice of practices, topic and type of prescription makes us confident in the findings of this analysis. However, clinical IT systems contain minimal data on GP characteristics and data about practices are restricted to structural characteristics rather than internal organisation, which limits the extent to which we can explain the variation observed.

## Background

Prescribed drugs are the single most commonly used medical intervention. They deliver significant individual and population benefit, but are also a common source of harm. Approximately 6.5% of emergency hospital admissions in the UK are caused by adverse drug events (ADEs),^1^ ^2^ and ADEs were the reason for an estimated 4.3 million ambulatory care consultations and over 100 000 hospital admissions in the USA in 2005.^3^ Commonly implicated drugs include antiplatelet agents such as aspirin, non-steroidal anti-inflammatory drugs (NSAIDs), anticoagulants, blood pressure lowering drugs and hypoglycaemic drugs,^2^ with deaths after admission most frequently due to antiplatelet drugs and NSAIDs.^1^ A large proportion of ADEs are preventable, but it is unclear whether risk varies between individual clinicians and healthcare settings.^4^

Many healthcare safety failures are largely attributable to shortcomings in the environment that individuals work in.^5^ It is not surprising that individuals do not wash their hands if there are no facilities for doing so, and fixing such system problems is critical. However, even with interventions to create better systems, hand washing remains highly variable between individuals.^6^ In some circumstances, the actions of a few individuals is an important determinant of system performance with, for example, 3% of Australian doctors accounting for 49% of complaints that were escalated to regional or federal ombudsmen.^7^ The appropriate balance between system and individual intervention to improve safety is therefore likely to depend on how strongly systems influence individual action and whether variation in process or outcome is determined more at the system or individual level.

There have been a small number of studies of primary care prescribing which have used robust statistical techniques such as multilevel modelling to examine variation,^4^ ^8–14^ but only two of these studies examined variation at multiple levels of the healthcare system. Both found that variation between clinics and variation between individual physicians were important determinants in variation in prescribing for osteoporosis and adherence to statin guidelines,^14^ ^11^ suggesting that individual action and practice culture both are important influences on prescribing. Further evidence that practice culture matters is provided by an analysis of adherence to three prescribing guidelines which found that practices tended to adhere (or not) to guidelines generally rather than adhering to some but not others, consistent with prescribers’ decisions to follow prescribing guidelines being significantly influenced by the wider ‘therapeutic traditions’ in the practice they work in.^13^

We have previously shown that high-risk prescribing is common in UK primary care, with an approximately fourfold variation between practices after accounting for casemix.^4^ However, it is unclear whether the observed variation between practices is mainly due to some practices having a particularly risky prescriber in them (an individual clinician or ‘bad apple’ problem) or is because clinicians in the same practice tend to prescribe in similar ways (a practice culture or ‘spoiled barrel’ problem). Understanding such variation is important to inform how best to target safety improvement.^15^ To the best of our knowledge, no study has examined how high-risk prescribing varies between individual prescribers and practices,^16^ which is the aim of this analysis.

## Methods

### Data set

Data were extracted from 38 Scottish general practices via the University of Aberdeen Primary Care Clinical Informatics Unit. We deliberately restricted analysis to these 38 practices because we considered that high-quality data recording was required for an analysis at GP level, and all these practices contributed to an NHS Scotland national morbidity recording data set and had therefore received training in, and financial support for, high-quality data recording. Like almost all UK general practices, these practices all used an electronic medical record which included data on morbidity (recorded as Read Codes, which are the universally used coding system for this purpose in the UK), demography and prescribing. Patients are only allowed to be registered with one general practice at a time, and that practice has responsibility for all community prescribing including that recommended by hospital specialists who only ever directly prescribe a small number of drugs for community use such as some cytotoxic agents.

We chose to focus on oral NSAID prescribing because it is a common cause of harm, and because the decision to initiate NSAIDs is almost completely attributable to the prescribing GP. This is not the case for many other high-risk drugs prescribed such as oral methotrexate in rheumatoid arthritis or antipsychotics in older people with dementia, where specialists frequently recommend initiation. Although the GP who acts on that recommendation takes legal responsibility for the prescription, attribution of the decision to the GP alone is clearly not straightforward. During initial data exploration, we established that the electronic medical record did not record the name of the doctor printed on ‘repeat’ prescriptions (those authorised for reissue by receptionists). We therefore focused on examining initiation of high-risk oral NSAID prescriptions, since initial analysis showed that clinician identifiers were reliably recorded in this context.

### Outcome and explanatory variables

The outcome examined was the issuing of a one-off oral NSAID prescription to an individual particularly vulnerable to NSAID ADEs who had not had an oral NSAID prescription in the 12 months before each encounter examined. Oral NSAIDs were defined as all oral preparations of drugs listed in British National Formulary (BNF) chapter 10.1.1 (which does not include aspirin).^17^ Patients were defined as particularly vulnerable to NSAID ADEs at the time of the encounter if one or more of five criteria was present: aged 75 years and older on or before the encounter date; Read Code for peptic ulcer ever recorded on or before the encounter date; Read Code for heart failure ever recorded on or before the encounter date; co-prescribed aspirin or clopidogrel at the time of the encounter; or co-prescribed an oral anticoagulant at the time of the encounter (warfarin, acenocoumarol or phenindione which were the only available drugs at the time).^18^ ^19^ To ensure that the NSAID was truly prescribed to a patient taking a relevant drug that increased bleeding risk, co-prescription was defined either as the NSAID being prescribed on the same day as aspirin, clopidogrel or an oral anticoagulant, or the aspirin, clopidogrel or oral anticoagulant being prescribed in the 84 days before *and* the 84 days after the NSAID prescription. NSAID prescribing for these individuals was clearly stated as risky and to be avoided in the March 2005 edition of the British National Formulary which is the most commonly used source of drug advice by UK prescribers. We therefore assumed that the potential risk of NSAID use in such patients was widely known in the time period examined.^17^

An encounter between a patient and a GP was eligible for analysis if it occurred between 1 January 2006 and 31 December 2006 at a time when the patient was permanently registered with the practice *and* the patient was particularly vulnerable to NSAID ADEs at the time of the encounter *and* had not had an NSAID prescribed in the year before the encounter. We chose to use 2006 because that year had the most practices available who were eligible for the study as described above. Encounters could be in a range of different contexts, including face to face in normal surgery, on home visits or on the telephone. An individual patient could therefore only have high-risk NSAID initiation once during the 1 year study period, and their encounters ceased to be eligible for inclusion after that NSAID initiation.

Explanatory variables were at the encounter, GP or patient level. Available data at the encounter level were the encounter type (normal surgery, telephone consultation or unknown/other), the number of risk factors for NSAID ADEs that a patient had at the time of the encounter, whether the patient had a ‘relevant diagnosis’ recorded in the encounter (defined as a Read Code from chapter N musculoskeletal conditions, chapter R ill-defined conditions/working diagnoses, chapter S injuries and poisoning, and chapter 1 history/symptoms, used primarily to account for case mix variation between general practitioners (GPs)). We additionally fitted variables which were characteristics of the patient having the encounter including: sex, age, socioeconomic status (measured by quintiles of postcode derived Carstairs Score^20^) and number of active repeat drugs at the start of 2006 as a measure of overall morbidity and resource use.

Only two explanatory variables were available at GP level: GP sex (recorded in the original data) and the number of encounters each GP had with patients at risk during the year (calculated from the encounter data and grouped into quartiles). At practice level, data on structural characteristics were available, namely the number of registered patients (listsize, grouped into quartiles), practice remoteness (three aggregated categories of the Scottish Executive Urban-Rural Classification—urban, accessible (≤30 min’ drive-time to an urban area), and remote (≤60 min)), whether or not the practice was accredited for postgraduate training of GPs, whether or not the practice was a dispensing practice, and whether or not the practice holds a General Medical Services contract (the standard national contract) or a locally specified contract.

### Statistical methods

In the UK, patients are only allowed to be permanently registered with one practice at a time, but since registration is with the practice rather than an individual GP, they can and do see multiple GPs. The outcome being examined happens in encounters between GPs and patients, but patients typically encounter several GPs and GPs encounter many patients. This means that there is no neat hierarchical clustering of patients with GPs within practices. We explored using several models of varying complexity, including models where encounters were cross-classified between GPs and patients (GPs have encounters with multiple patients, and patients have encounters with multiple GPs, all clustered within practices), but these more complex models would not converge. Consequently, the analysis presented here is a three-level hierarchical model of encounters clustered within GPs clustered within practices, which is an approximation to the reality but a useful model for this purpose. Since the outcome is binary (a high-risk NSAID is either initiated in the encounter or not), multilevel logistic regression models with random slopes at GP and practice level were fitted. Assumptions about the normality of higher level residuals were checked graphically. The intraclass correlation (ICC) coefficient at GP and practice level was calculated in the empty model with no explanatory variables to estimate the proportion of variation in outcome that was attributable to variation between GPs and variation between practices. Encounter level explanatory variables were fitted first and multilevel univariate and adjusted ORs of associations between the explanatory variables and high-risk NSAID initiation calculated.

Variation between GPs and between practices was then re-examined after adjusting for differences in encounter/patient characteristics. GPs and practices with statistically significantly higher or lower high-risk NSAID prescribing were identified using the GP and practice level residuals. Median ORs at GP and practice level were calculated to provide an estimate of variation on the same scale as the fixed effects ORs.^21^ The median OR at *GP* level can be interpreted as the median difference in the odds of high-risk NSAID initiation if the same patient was to randomly encounter two different GPs in the same practice, and the median OR at *practice* level as the median difference in the odds of high-risk NSAID initiation if the patient was to randomly encounter two different GPs from different practices. Finally, associations between GP and practice characteristics and high-risk prescribing were examined.

Initial data management and analysis was carried out in SPPS V.21, and multilevel modelling in Stata IC V.11. The NHS National Research Ethics Service had previously approved the anonymous use of these data for research purposes; therefore, this study did not need individual ethics approval.

## Results

The data set for analysis consisted of 181 010 encounters in the calendar year 2006 between 26 539 eligible patients and 398 GPs in 38 practices. Each patient had a mean of 6.8 (95% CI 6.7 to 6.9) eligible encounters, and each GP a mean of 455 (95% CI 414 to 496) encounters with eligible patients. The median age of included patients was 76 years (IQR 67–82), and 14 062 (53.0%) were women. At the start of the year, the median number of risk factors per patient for NSAID ADEs was 1 (range 1–5, IQR 1–2) and the median number of repeat drugs taken was 4 (range 0–30, IQR 1–6). A total of 239 (60.1%) GPs were male.

**Figure 1:**
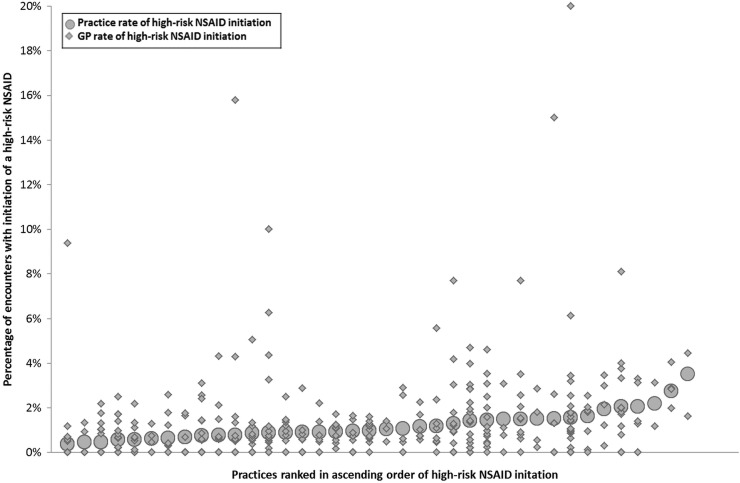
Variation in high-risk NSAID initiation between practices (large circles) and between GPs within practices (small diamonds) (133 GPs have zero rates, so these and some other plotted GP points overlap). NSAIDs, non-steroidal anti-inflammatory drugs; GP, general practitioners.

During the year of observation, a high-risk NSAID was initiated in 1953 of the 181 010 eligible encounters (1.08% of encounters, 95% CI 1.03% to 1.13%). Put another way, 1953 of 26 539 patients particularly vulnerable to NSAID ADEs had a high-risk NSAID initiated (7.4% of patients, 95 CI 7.1% to 7.7%). At practice level, the high-risk prescribing rate varied from 0.37% to 3.50% of encounters (median 1.01%, IQR 0.76% to 1.51%), and at GP level the rate varied from 0 to 20.0% of encounters (median 0.68%, IQR 0% to 1.50%). Figure 1 shows how the actual practice rates and individual GP rates varied. There is a visual impression of substantial variation between practices and between GPs in the same practice, although it is noteworthy that 133 (33.4%) GPs had no high-risk NSAID prescribing in the year examined.

Table 1 shows associations between encounter/patient and GP characteristics and high-risk NSAID initiation. In the adjusted model, the most strongly associated variable was whether or not a relevant diagnosis had been recorded for the encounter (OR 7.03, 95% CI 6.32 to 7.82). Compared to normal surgery encounters, high-risk NSAID initiation was less common in telephone encounters (OR 0.68, 95% CI 0.52 to 0.89) and other/unknown encounters (OR 0.34, 95% CI 0.29 to 0.42). High-risk NSAID initiation was less likely with increasing numbers of risk factors for NSAID associated ADEs (OR 0.61. 95% CI 0.49 to 0.76, in those eligible for ≥3 indicators vs those eligible for one). Initiation was less common in the oldest two groups compared to the youngest (80 years and over vs under 50 years OR 0.59, 95% CI 0.49 to 0.72). Encounters with people taking 11 or more repeats had half the odds of resulting in high-risk NSAID initiation than those with people with no active repeat drugs (OR 0.51, 95% CI 0.39 to 0.68). Male GPs were more likely to initiate a high-risk NSAID (OR 1.73, 95% CI 1.39 to 2.16, for male GPs compared to female GPs).

**Table 1:** Multilevel adjusted associations (only statistically significantly associated variables shown*)

Encounter and GP characteristics (number of encounters) n=181 010 encounters, 398 GPs, 38 practices	% (95% CI) of encounters with high-risk NSAID initiation	Multilevel univariate OR (95% CI)	Multilevel adjusted OR (95% CI)
Encounter type			
Normal surgery (n=133 614)	1.33 (1.27 to 1.40)	1	1
Telephone (n=16 855)	0.46 (0.27 to 0.45)	0.26 (0.20 to 0.33)	0.68 (0.52 to 0.89)
Unknown/other (n=30 541)	0.36 (0.30 to 0.44)	0.31 (0.25 to 0.37)	0.34 (0.29 to 0.42)
Indicators triggered at encounter date			
1 (n=99 389)	1.38 (1.30 to 1.46)	1	1
2 (n=61 404)	0.79 (0.72 to 0.86)	0.58 (0.52 to 0.64)	0.81 (0.73 to 0.91)
≥3 (n=20 217)	0.48 (0.38 to 0.58)	0.35 (0.28 to 0.42)	0.61 (0.49 to 0.76)
Relevant diagnosis at encounter			
No (n=127 984)	0.40 (0.37 to 0.44)	1	1
Yes (n=53 026)	2.72 (2.56 to 2.86)	7.12 (6.48 to 7.97)	7.03 (6.32 to 7.82)
Patient age			
<50 years (n=8893)	2.18 (1.85 to 2.51)	1	1
50–59 years (n=10 600)	1.98 (1.70 to 2.26)	0.97 (0.79 to 1.18)	1.07 (0.87 to 1.32)
60–69 years (n=30 991)	1.41 (1.28 to 1.55)	0.68 (0.57 to 0.81)	0.93 (0.77 to 1.12)
70–79 years (n=64 502)	1.05 (0.96 to 1.13)	0.49 (0.42 to 0.58)	0.74 (0.62 to 0.89)
80+ years (n=66 024)	0.66 (0.60 to 0.72)	0.31 (0.26 to 0.37)	0.59 (0.49 to 0.72)
No of repeat drugs			
0 (n=24 051)	1.76 (1.58 to 1.94)	1	1
1–2 (n=31 435)	1.39 (1.25 to 1.52)	0.74 (0.64 to 0.85)	0.86 (0.74 to 0.99)
3–4 (n=42 589)	0.85 (0.75 to 0.95)	0.58 (0.50 to 0.66)	0.73 (0.63 to 0.84)
5–6 (n=36 075)	0.77 (0.65 to 0.88)	0.46 (0.40 to 0.54)	0.61 (0.52 to 0.72)
7–8 (n=23 926)	0.77 (0.65 to 0.88)	0.38 (0.35 to 0.50)	0.61 (0.50 to 0.73)
9–10 (n=12 897)	0.70 (0.55 to 0.85)	0.38 (0.30 to 0.48)	0.55 (0.43 to 0.71)
11+ (n=10 037)	0.62 (0.46 to 0.78)	0.32 (0.24 to 0.42)	0.51 (0.39 to 0.68)
GP sex			
Women (n=159 GPs, 67 615 encounters)	0.68 (0.53 to 0.83)	1	1
Men (n=239 GPs, 113 395 encounters)	1.32 (1.17 to 1.46)	1.82 (1.44 to 2.31)	1.73 (1.39 to 2.16)

*Patient sex, deprivation, GP number of encounters, and all practice variables (list size, rurality, contract type, training status, dispensing) were examined but were not significantly associated and therefore not included.

GP, general practitioners; NSAIDs, non-steroidal anti-inflammatory drugs.

The practice level, ICC in the empty model was 0.055 (95% CI 0.029 to 0.102) compared to 0.042 (95% CI 0.021 to 0.083) in the model adjusted for patient/encounter level variables (table 2). The GP level ICC in the empty model was 0.166 (95% CI 0.135 to 0.197) compared to 0.142 (95% CI 0.114 to 0.173) in the model adjusted for patient/encounter level variables. After adjustment for patient/encounter characteristics, approximately three times more variation in high-risk NSAID initiation was attributable to variation between GPs (14.2%) than variation between practices (4.2%).

**Table 2:** Variation between practices and between GPs before and after inclusion of patient and GP characteristics*

	Intraclass correlation coefficient ICC (95% CI)	Median OR (95% CI)†
Empty model (no patient or GP characteristics included)		
Practice level	0.055 (0.029 to 0.102)	2.52 (2.15 to 3.09)
GP level	0.166 (0.135 to 0.197)	2.22 (2.00 to 2.50)
Patient model (only patient characteristics included)		
Practice level	0.042 (0.021 to 0.083)	2.28 (1.98 to 2.76)
GP level	0.142 (0.114 to 0.173)	2.06 (1.87 to 2.30)
Full model (patient and GP characteristics included)		
Practice level	0.031 (0.014 to 0.068)	2.14 (1.88 to 2.57)
GP level	0.131 (0.103 to 0.161)	1.98 (1.80 to 2.21)

*Included characteristics are those listed in table 1. Patient sex, deprivation, GP number of encounters, and all practice variables (list size, rurality, contract type, training status, dispensing) were examined but were not significantly associated and therefore not included.

†The median OR at GP level can be interpreted as the median difference in the odds of high-risk NSAID initiation if the same patient were to randomly encounter two different GPs in the same practice. The median OR at practice level can be interpreted as the median difference in the odds of high-risk NSAID initiation if the patient were to randomly encounter two different GPs from different practices (but should be interpreted in terms of how different it is from the median OR at GP level since it includes variation between GPs as well as between practices. GP, general practitioners; ICC, intraclass correlation; NSAIDs, non-steroidal anti-inflammatory drugs.

The median OR at GP level was 2.22 (95% CI 2.00 to 2.50) in the empty model and accounting for patient and encounter characteristics reduced it slightly to 2.06 (95% CI 1.87 to 2.30), while the median OR at practice level was 2.52 (95% CI 2.15 to 3.09) in the empty model and accounting for patient and encounter characteristics reduced it to 2.28 (95% CI 1.98 to 2.76). On average, the likelihood of high-risk NSAID initiation therefore varied approximately twofold if a patient was to randomly encounter two different GPs in the same practice, and only slightly more if a patient was to randomly encounter two GPs working in different practices, again indicating that the greatest variation is between GPs.

**Figure 2:**
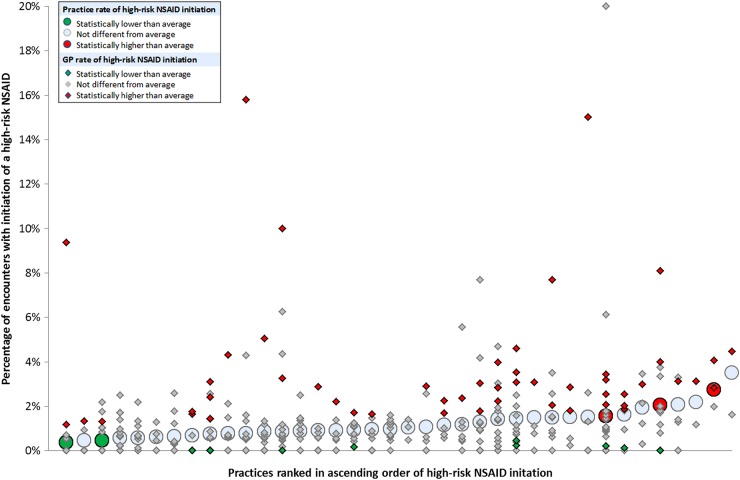
Variation in high-risk NSAID initiation between practices (large circles) and between GPs within practices (small diamonds). (On the basis of the multilevel model after accounting for encounter and patient characteristics, green indicates GP or practice is statistically lower than average, red indicates GP or practice is statistically higher than average. 133 GPs have zero rates, so these and some other plotted GP points overlap). NSAIDs, non-steroidal anti-inflammatory drugs; GP, general practitioners.

After adjustment for patient and encounter characteristics, 3 of the 38 (7.9%) practices had statistically significantly higher than average rates of high-risk NSAID initiation, and 2 (5.3%) had statistically significantly lower rates. At GP level, 51 (12.8%) of 398 GPs had statistically significantly higher than average high-risk NSAID initiation, and 10 (2.5%) had statistically significantly lower rates. Figure 2 shows how these practices and GPs were distributed. GPs with higher or lower than average rates of high-risk NSAID initiation were distributed across the entire range of practices. Only 8/51 (15.7%) GPs with statistically significantly higher than average prescribing were in the three practices with statistically significantly higher than average prescribing, and only 369 (18.9%) of the high-risk NSAID initiations were in these three practices.

## Discussion

High-risk NSAID initiation in patients at particularly high risk of NSAID related ADEs occurred in 1.1% of eligible encounters in 2006. In the full multilevel model, high-risk NSAID prescribing was more likely to occur in normal surgery encounters and less likely to occur in encounters with patients with more risk factors for NSAID adverse drug effects, in encounters with older patients, and in encounters with patients prescribed more repeat drugs. These findings suggest that GPs generally perceived the NSAID prescribing examined to be risky because they were on average less likely to prescribe in people at higher risk of NSAID related ADEs. At GP level, male prescribers were more likely to initiate a high-risk NSAID than female GPs (OR 1.73) even after adjustment for casemix. None of the practice structural characteristics examined were associated with high-risk prescribing.

After accounting for encounter and patient characteristics, 4.2% of the variation in high-risk NSAID prescribing was attributable to variation between practices (similar to between-practice variation found in other studies^16^), and 14.2% to variation between GPs (at the upper end of between-physician variation found in other studies^16^). Variation between GPs was of similar magnitude to most of the individual characteristics (such as patient age and sex) examined, in that the odds of NSAID initiation in a patient randomly encountering two different GPs in the same practice varied twofold on average simply by virtue of seeing different GPs.

A strength of the study is that the use of data routinely recorded in GP electronic medical records makes it possible to examine actual practice on a large scale, but all such studies share limitations, notably the type and quality of the data recorded. In terms of type of data, we had virtually no information about the GPs except their sex, because nothing else is routinely recorded in clinical IT systems, and only limited information about practice structure but not internal organisation. Data quality depends on how practices use their clinical IT system, although all the practices in this analysis contributed to a national morbidity data set and had received training and financial support to maintain high quality data. However, the use of such practices does potentially limit the generalisability of the findings since we do not know how their prescribing compares with other practices. Finally, several NSAIDs are available from pharmacists to buy over the counter which cannot be accounted for in GP data, although neither GPs nor practices are directly responsible for such use. We therefore believe that this analysis of high-risk NSAID initiation is valid (although extending to repeat prescriptions is not possible with current UK data, and extending to other drugs would have to account for the difficulties of attributing the decision to initiate which will sometimes be made by a specialist). The study is therefore better at quantifying the extent of variation (which was the primary objective of our study) than explaining the variation observed. For example, we do not know whether some GPs were not aware of the risks (a knowledge problem potentially amenable to relatively simple educational interventions) or had higher risk tolerance (which might be associated with GP sex, eg, but where more intensive interventions would most likely be needed to change behaviour).

Studies of variation at multiple levels of healthcare systems are relatively rare, with only 12 identified by a recent systematic review of 39 studies using multilevel modelling or other appropriate techniques to examine variation.^16^ In these studies, variation between physicians was usually greater than variation between the institutions or areas that those physicians worked in. However, patterns of variation appear to depend on the extent to which the outcome examined is directly controlled by individual doctors or more reliant on the organisation of care by the practices or hospitals those doctors work in. For example, in a study examining diabetes care, between-physician variation in blood pressure measurement was larger than between-hospital variation, whereas between-hospital variation in eye screening was larger than between-physician variation.^22^ Roberts *et al*^23^ elegantly show that variation in patient satisfaction for outcomes controlled by the practice, such as cleanliness or building access, was largely at practice level, whereas variation for consultation outcomes such as communication was much larger at GP level. The authors observed that practices with high overall satisfaction rates rarely or never had a low performing GP in them, and that practice satisfaction could therefore be used as a screening tool to identify practices where measurement at GP level would be helpful. In contrast, our study found that GPs with significantly higher and lower rates of high-risk NSAID initiation were distributed across the entire range of practice rates, and focusing only on higher risk practices would miss most higher risk GPs and most high-risk prescribing.

Key implications are that measurement of high-risk prescribing at GP level is not routinely feasible using electronic data in the UK because electronic data do not reliably record who signed the paper prescription, which is a particular issue for ‘repeat’ prescriptions authorised for regular reissue. For new prescriptions, electronic data are reasonably reliable, but attribution may still be problematic because, although the GP who signs the prescription takes legal responsibility for it, many drugs are initiated on specialist recommendation. Measurement at practice level can identify practices with higher than average prescribing, but most (84.3%) GPs with significantly raised high-risk prescribing and most (81.5%) patients initiated on a high-risk prescription are not in these practices. Although some practices with particularly high rates of high-risk prescribing may need individual support, primary care prescribing safety improvement will therefore most likely have to target all practices. This could take the form of regular feedback of practice rates with facilitation of review of patients receiving high-risk prescribing and examination by the practice of internal variation between GPs to inform an appropriate practice response.^24^ Routine measurement at GP level will require universal adoption of true electronic prescribing where the ‘signing’ of the prescription is done with a unique identifier, such as the General Medical Council registration number in the UK.

Finally, although this study has quantified variation in high-risk prescribing between practices and between GPs, the data available do not allow much exploration of factors explaining such variation. The only GP or practice characteristic associated with high-risk prescribing was GP sex, with male GPs being more likely to initiate a high-risk NSAID than female GPs, but whether this relates to a different casemix beyond what could be controlled for in this study, or greater knowledge gaps about the risk and benefit of drugs, or greater tolerance of risk by men is uncertain. Research is needed to better understand such factors and how these influence prescribing decisions. For example, although high-risk NSAID initiation was less common in people with multiple risk factors for NSAID ADEs implying that GPs were generally aware of increasing risk, actually understanding how risk and benefit are balanced in decision-making using appropriate qualitative (eg, observation and ‘think-aloud’ interviewing) or quantitative (eg, discrete choice experiments) methods would be very useful. Similarly, it is unclear whether practices or GPs have a general tendency to be high-risk prescribers across multiple measures or whether most practices have one or more areas where they are different from average. Finally, there are relatively few studies of interventions to reduce primary care high-risk prescribing, with the best evidence to date for pharmacist-led interventions,^25^ ^26^ although other studies of GP-led interventions are in progress.^27^ ^28^ Returning to the original question, the findings suggest that high-risk prescribing is more of a ‘bad apple’ than a ‘spoiled barrel’ problem, but improvement is likely to require the whole crop of prescribers to take professional responsibility for high-risk prescribing in their practice and to work collaboratively to minimise preventable harm from drug therapy.
